# Association between SMAD3 gene polymorphisms and osteoarthritis risk: a systematic review and meta-analysis

**DOI:** 10.1186/s13018-018-0939-2

**Published:** 2018-09-12

**Authors:** Jian-qiao Hong, Yang-xin Wang, Si-hao Li, Guang-yao Jiang, Bin Hu, Yu-te Yang, Jia-hong Meng, Shi-gui Yan

**Affiliations:** Department of Orthopaedic Surgery, The Second Affiliated Hospital, Zhejiang University School of Medicine, No.88 Jiefang Road, Hangzhou, 310009 People’s Republic of China

**Keywords:** SMAD3, Polymorphism, Osteoarthritis, Meta-analysis

## Abstract

**Objective:**

Several studies have been performed to investigate the association between SMAD3 gene polymorphism and osteoarthritis (OA), but the results were inconclusive. This study aims to determine whether SMAD3 polymorphism is associated with risk of OA.

**Method:**

A comprehensive literature search in PubMed, Embase, and ISI Web of Science for relevant studies was performed. After extracting data from eligible studies, we chose the fixed or random effect model according to the heterogeneity test. Estimation of publication bias and sensitivity analysis were conducted to confirm the stability of this meta-analysis.

**Results:**

In total, 10 studies from 6 articles with 5093 OA patients and 5699 controls were enrolled in this meta-analysis. The combined results revealed significant association between SMAD3 rs12901499 polymorphism and the risk of OA (allele model: OR 1.21, 95% CI 1.07–1.38). Subgroup analysis revealed that G allele increased the risk of OA in Caucasians, but not in Asians (allele model: Caucasians: OR 1.31, 95% CI 1.18–1.44; Asians: OR 1.24, 95% CI 0.95–1.61). And the pooled results revealed significant association between SMAD3 rs12901499 polymorphism and both knee and hip OA (knee OA: OR 1.18, 95% CI 1.04–1.34; hip OA: OR 1.31, 95% CI 1.18–1.44).

**Conclusion:**

The current meta-analysis revealed that the G variant of SMAD3 rs12901499 polymorphism increased the risk of OA in Caucasians. Further well-designed studies with larger sample size in different ethnic populations are required to confirm these results.

**Electronic supplementary material:**

The online version of this article (10.1186/s13018-018-0939-2) contains supplementary material, which is available to authorized users.

## Background

Osteoarthritis (OA), a late-onset musculoskeletal disease in the elder, is featured by the gradual degradation of articular cartilage with further lesion to the synovium, subchondral bone, or the other joint tissues. The osteoarthritis could cause chronic joint pain with swelling and restricted range of motion through the pathologic process including narrowing of joint space and osteophyte formation [1–3]. It was estimated that over 15% of the population suffered from OA, and the number tends to be doubled by 2020 due to the increasing elder population [4, 5]. Though the mechanism of osteoarthritis still has not been fully clarified, a large number of risk factors have been reported, including age, sex, obesity, trauma in the joint, environmental factors, and genetic factors [3, 6, 7].

The SMAD3 (SMAD family member 3) gene, located on chromosome 15q22.33, acts a critical role in the joint homeostasis [8, 9]. It is known as a downstream mediator in the TGF-B (transforming growth factor-b) signaling pathway which plays a key role in anabolism of chondrocytes [9]. The genetic variations of TGF-B signaling have been reported significant relationship to the OA [10]. In TGF-β signaling pathway, SMAD3 translocates into the nucleus and regulates target gene transcription and produces the phenotype in cartilage by interaction with DNA and transcription factors [11]. Several studies have been performed to investigate the association between SMAD3 polymorphisms and OA susceptibility, but the results remained unclear. Valdes [12] revealed that the SMAD3 gene rs12901499 polymorphism was associated with hip and knee OA in European populations. However, the results reported by other subsequent studies remain inconsistent and inconclusive [13–17]. In this study, we therefore performed a meta-analysis to evaluate whether the SMAD3 gene polymorphisms are associated with the risk of OA.

## Materials and methods

### Literature search strategy

We conducted a comprehensive literature search using the electronic databases PubMed, Embase, and ISI Web of Science for relevant studies published in English (last search was updated on April 10, 2018). The search strategy was based on the following keywords: (“SMAD3” OR “SMAD family member 3”) AND (“polymorphism” OR “variant” OR “SNP”) AND (“osteoarthritis” OR “OA”). References of clinical trials and review articles were also searched manually for additional articles. All the literature search was performed according to the Preferred Reporting Items for Systematic Reviews and Meta-Analyses (PRISMA) guidelines (Additional file 1: Table S1) [18].

### Inclusion criteria

Two researchers screened the relevant investigations and further determined the eligible studies which met the following inclusion criteria: (1) case-control or cohort design; (2) evaluating the association between SMAD3 polymorphisms and knee/hip OA susceptibility; (3) patients with OA were diagnosed based on clinical manifestation and radiographic findings, or received total joint arthroplasty because of primary OA; and (4) enough data on genotype or allele frequency for calculation of odds ratio (OR) and corresponding 95% confidence interval (CI). The animal model research, review, case reports, or the studies without sufficient data were excluded. If several articles reported findings for repeated study populations, we only selected the most recent study or the one with the largest sample size.

### Data extraction

For each eligible study, two independent investigators extracted the following data: first author’s name, publication year, country and ethnicity of study population, study design, OA sites, sample size, demographics of enrolled subjects, genotyping method, studied polymorphisms, and genotype distributions.

### Quality assessment

According to the Newcastle-Ottawa Quality Assessment Scale (NOS) [19], the quality score of each study was based on three categories: selection (4 items, 1 point each), comparability (1 item, up to 2 points), and exposure/outcome (3 items, 1 point each). Each study scored from 0 point (worst) to 9 points (best), and scored 6 or less were classified as low quality, whereas studies scoring 7 or higher were defined as high quality.

### Statistical analysis

All statistical analyses were conducted with STATA version 12.0 (STATA Corporation, College Station, TX, USA), and *p* value < 0.05 was considered significant except for the *I*^2^ statistic. To assess the correlation between SMAD3 polymorphisms and OA susceptibility, we calculated pooled ORs with 95% CI and analyzed five genetic models: allele model, dominant model, recessive model, homozygous model, and heterozygous model.

Heterogeneity between studies was measured using *Q* and *I*^2^ statistics [20]. If *I*^2^ > 50% and *p* value of *Q* statistic < 0.10, the DerSimonian-Laird random effect model was applied to calculate pooled ORs and 95% CIs [21]. Otherwise, a fixed effect model was used as the pooling method [22].

Subgroup analysis was conducted by ethnicity, OA site. Sensitivity analysis was also performed by removing individual study sequentially in order to evaluate the stability of pooled results. We evaluated the publication bias by funnel plot and Egger’s regression test.

## Results

### Study selection

**Fig. 1:**
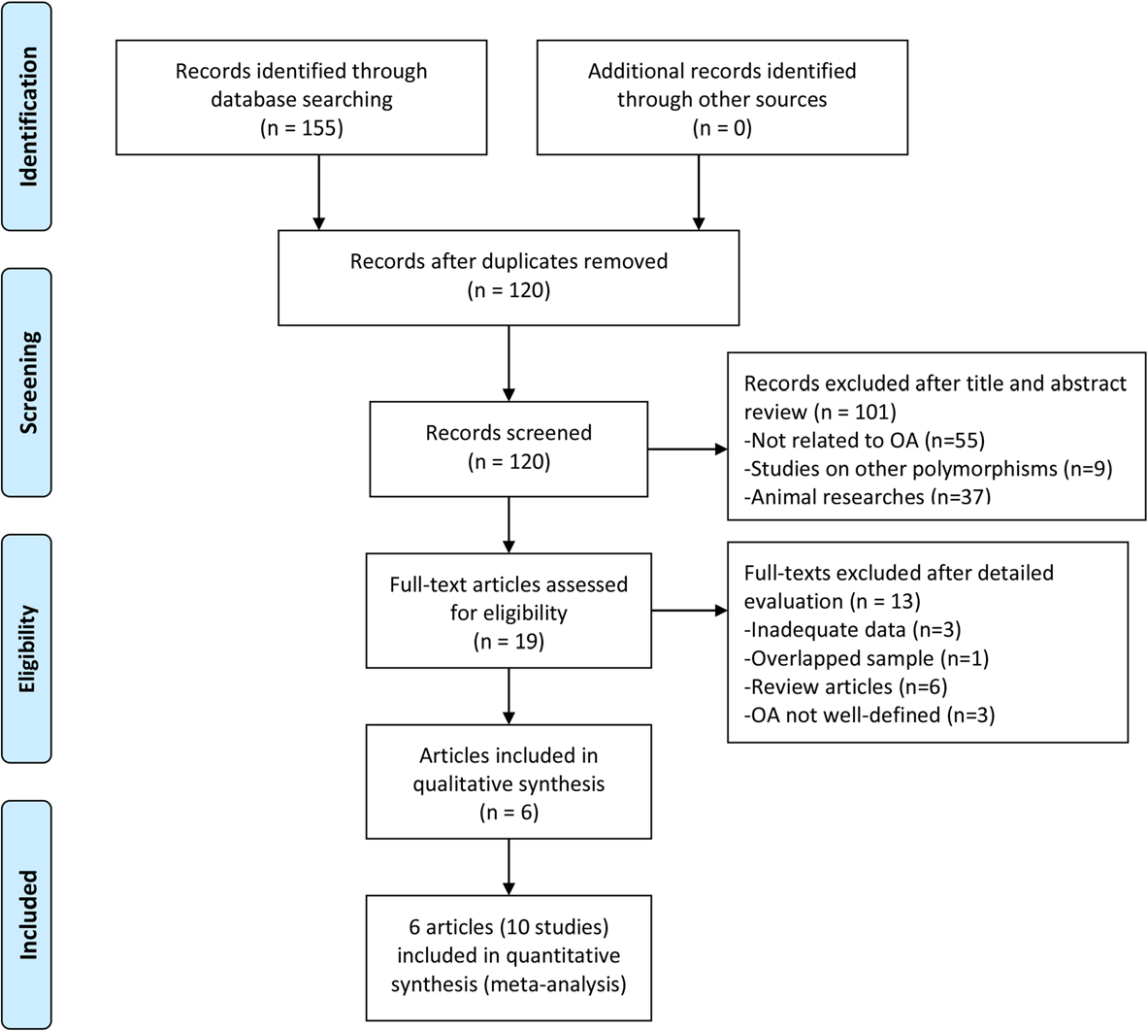
Flow chart of the study selection process

Figure 1 presented the selection process and reasons for exclusion. One hundred fifty-five articles were retrieved totally from a systematic literature search, 35 articles were removed because of duplications, and 101 articles were excluded after review of title and abstract; only 19 full-text articles remained for further evaluation. Subsequently, 4 articles were excluded because of inadequate data, 1 article was excluded due to overlapped samples, and 8 review articles were removed; 3 articles studied generalized OA, temporomandibular joint OA, and spinal OA, respectively, and were not included because the OA patients included by those studies were not well defined.

Finally, 6 articles met our inclusion criteria [12–17]. One article provided by Valdes included 5 different study populations; these 5 studies were analyzed independently. Therefore, 10 independent studies from 6 articles were included in our meta-analysis.

### Characteristics of included studies

Table 1 shows the main characteristics of included studies, and Table 2 presents the genotype and allele distributions of the SMAD3 rs12901499 polymorphism. A total of 5093 OA patients and 5699 controls were included in this study, which involved 5 Caucasian and 5 Asian populations. Patients diagnosed with OA were recruited according to clinical and radiographic results or ascertained by total joint arthroplasty. The genotype distribution of the control group showed conformation to Hardy-Weinberg equilibrium in all the included studies. As for the sites of OA, 9 studies examined knee OA, and 4 studies examined hip OA. Regarding the NOS scale, the quality of all the included studies was fairly high (Additional file 2: Table S2). Eventually, all the 10 studies from the articles as stated above were included in the meta-analysis for further research.

**Table 1:** Characteristics of included studies

Study	Year	Country	Ethnicity	Design	OA site	Genotyping method	Sample size (F/M)		HWE	NOS
							OA	Control		
Valdes-Discovery set	2010	UK	Caucasian	CC	Knee, hip	Allele-specific PCR	477 (NA)	520 (NA)	Y	7
Valdes-Nottingham	2010	UK	Caucasian	CC	Knee, hip	Allele-specific PCR	2014 (NA)	733 (NA)	Y	7
Valdes-Chingford	2010	UK	Caucasian	Cohort	Knee, hip	Allele-specific PCR	317 (317/0)	488 (488/0)	Y	9
Valdes-Hertfordshire	2010	UK	Caucasian	Cohort	Knee	Allele-specific PCR	167 (NA)	867 (NA)	Y	8
Valdes-Estonia	2010	Estonia	Caucasian	Cohort	Knee	Allele-specific PCR	68 (NA)	449 (NA)	Y	8
Jiang	2013	China	Asian	CC	Knee	PCR-RFLP	232 (159/73)	236 (91/145)	Y	7
Su	2015	China	Asian	CC	Knee	PCR-RFLP	518 (328/190)	468 (261/207)	Y	7
Sharma	2017	India	Asian	CC	Knee	Allele-specific PCR	450 (230/220)	458 (234/224)	Y	8
Zhang	2018	China	Asian	CC	Knee	Allele-specific PCR	350 (99/251)	400 (110/210)	Y	8
Zhong	2018	China	Asian	CC	Hip	Allele-specific PCR	500 (260/240)	1080 (580/500)	Y	8

*CC* case-control, *F* female, *M* male, *NA* number of each gender not available, *HWE* Hardy-Weinberg equilibrium, *NOS* Newcastle-Ottawa Quality Assessment Scale

**Table 2:** Genotype and allele distributions of SMAD3 rs12901499 polymorphism in the included studies

Study	Genotype distribution						Allele distribution				Association findings
	OA			Control			OA		Control		
	AA	AG	GG	AA	AG	GG	A	G	A	G	
Valdes-Discovery set	NA	NA	NA	NA	NA	NA	419	635	489	551	G allele↑
Valdes-Nottingham	NA	NA	NA	NA	NA	NA	1788	2336	698	768	G allele↑
Valdes-Chingford	NA	NA	NA	NA	NA	NA	316	388	477	499	NS
Valdes-Hertfordshire	NA	NA	NA	NA	NA	NA	134	200	779	955	NS
Valdes-Estonia	NA	NA	NA	NA	NA	NA	67	79	481	417	NS
Jiang	47	141	25	114	83	23	235	191	311	129	G allele↑, GG genotype↑
Su	142	247	129	116	228	124	531	505	460	476	NS
Sharma	85	70	75	90	92	52	240	220	272	196	G allele↑, GG genotype↑
Zhang	82	173	91	81	202	111	337	355	364	424	GG genotype↓
Zhong	10	200	290	20	610	450	220	780	650	1510	G allele↑, GG genotype↑

*NA* data not available, *↑/↓* increase/decrease the risk of OA, *NS* not significant

### Association between SMAD3 rs12901499 polymorphism and OA susceptibility

**Fig. 2:**
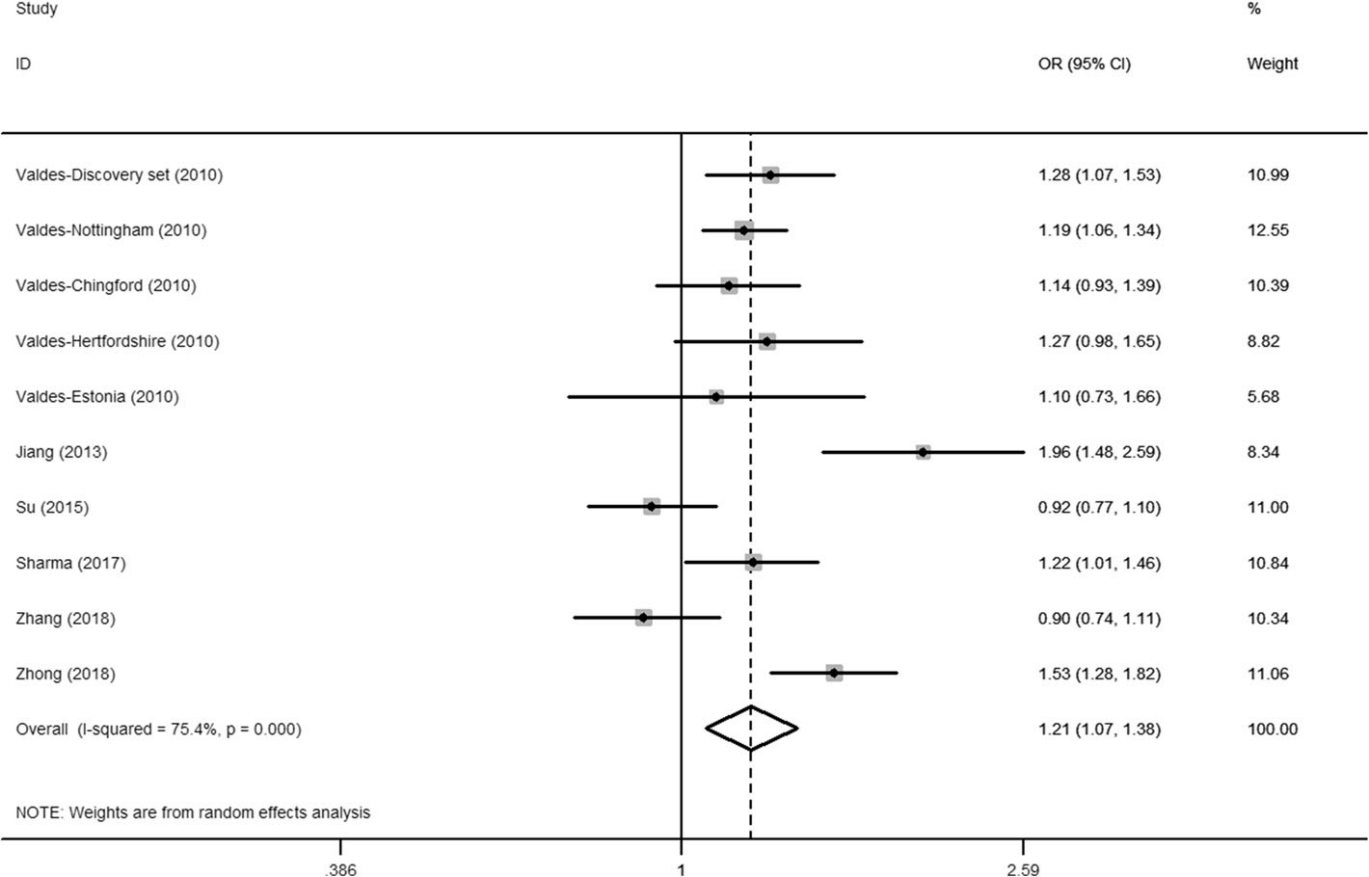
Meta-analysis for the association between SMAD3 rs12901499 polymorphism and OA risk. The squares and horizontal lines denote the ORs and 95% CIs of individual studies, and the size of the squares corresponds to the study-specific weight. The hollow diamond denotes the pooled OR and 95% CI

Table 3 and Fig. 2 summarize the meta-analysis results on the association between *SMAD3 rs12901499* polymorphism and risk of OA. Because genotype distribution data was not reported by Valdes, only allele model was analyzed in the overall population and European population. Overall, the combined results revealed a significant association between SMAD3 rs12901499 polymorphism and the risk of OA (allele model: OR 1.21, 95% CI 1.07–1.38); (Table 3; Fig. 2).

**Table 3:** Pooled results on the association between SMAD3 rs12901499 polymorphism and OA risk

Genetic model	Sub-group	No. of studies	Test of association		Statistical model	Test of heterogeneity	
			OR (95% CI)	*p*		*I*^2^ (%)	*p*
Allele model (G vs. A)	Overall	10	1.21 (1.07–1.38)	0.003	Random	75.4	0.000
	Ethnicity						
	Asian	5	1.24 (0.95–1.61)	0.115	Random	88.7	0.000
	Caucasian	5	1.20 (1.11–1.31)	0.000	Fixed	0.0	0.891
	OA site						
	Knee OA	9	1.18 (1.04–1.34)	0.011	Random	67.1	0.002
	Hip OA	4	1.31 (1.18–1.44)	0.000	Fixed	39.9	0.172

When we divided the participants according to ethnicity, G allele was associated with increased risk of OA in Caucasian rather than in Asians (allele model: Caucasian: OR 1.31, 95% CI 1.18–1.44; Asian: OR 1.24, 95% CI 0.95–1.61) (Table 3). What is more, stratification by OA site showed that SMAD3 rs12901499 polymorphism was significantly associated with the risk of both knee OA and hip OA (knee OA: OR 1.18, 95% CI 1.04–1.34; hip OA: OR 1.31, 95% CI 1.18–1.44) (Table 3).

For Asian subgroup, other genetic models were also analyzed (allele model: OR 1.28, 95% CI 0.87–1.88; dominant model: OR 1.18, 95% CI 0.70–2.00; recessive model: OR 1.29, 95% CI 0.90–1.86; homozygote model: OR 1.21, 95% CI 0.83–1.77; heterozygote model: OR 1.06, 95% CI 0.56–1.99) (Table 4).

**Table 4:** Pooled results on the association between SMAD3 rs12901499 polymorphism and OA risk in Asians

Genetic model	No. of studies	Test of association		Statistical model	Test of heterogeneity	
		OR (95% CI)	*p*		*I*^2^ (%)	*p*
Allele model (G vs. A)	5	1.28 (0.87–1.88)	0.203	Random	90.1	0.000
Dominant model (GG+AG vs. AA)	5	1.18 (0.70–2.00)	0.529	Random	90.2	0.000
Recessive model (GG vs. AG+AA)	5	1.29 (0.90–1.86)	0.166	Random	85.3	0.000
Homozygote model (GG vs. AA)	5	1.21 (0.83–1.77)	0.324	Random	70.6	0.009
Heterozygote model (AG vs. AA)	5	1.06 (0.56–1.99)	0.857	Random	92.3	0.000

### Sensitivity and publication bias analysis

**Fig. 3:**
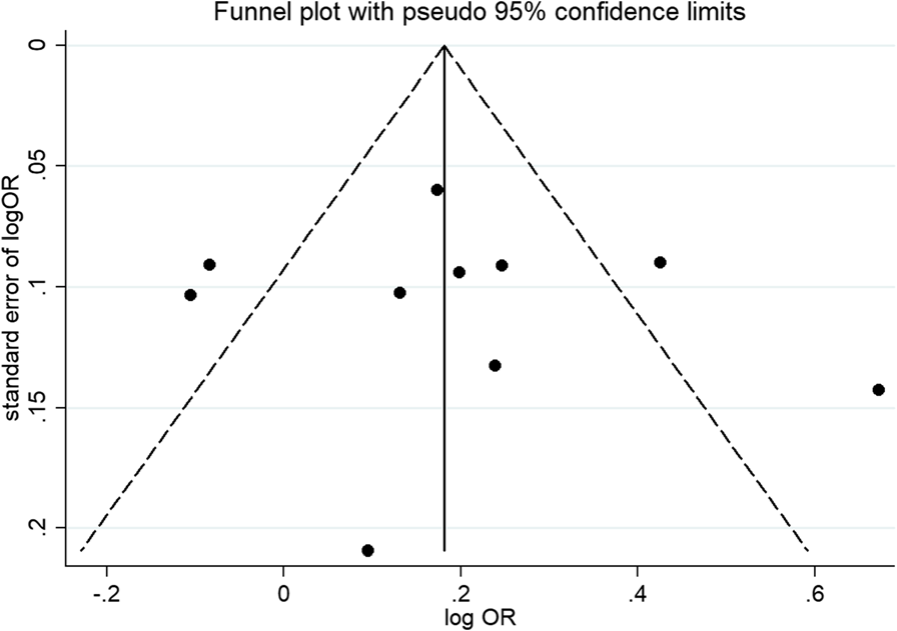
Funnel plot

**Fig. 4:**
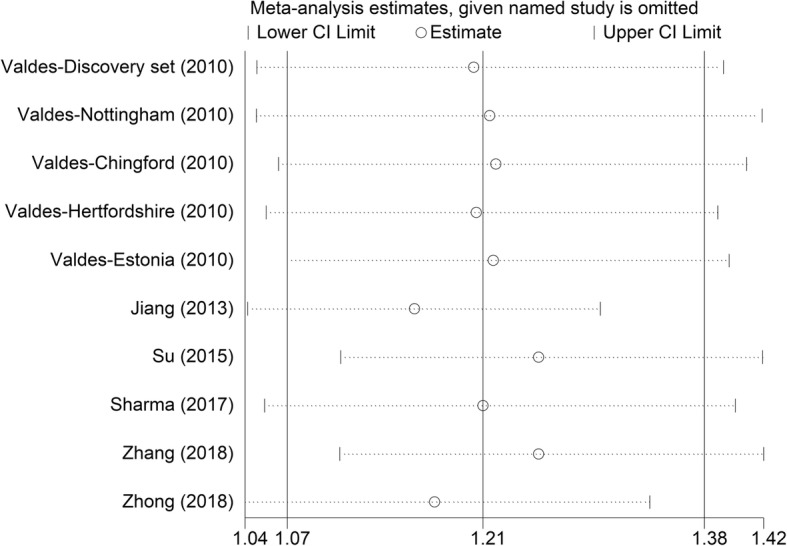
Sensitivity analysis

With the aid of funnel plots and Egger’s test (Table 3; *p* egger = 0.692), we find no significant publication bias (Fig. 3). Furthermore, by using sensitivity analysis (Fig. 4), the combined results remained stable after removing individual studies. The robustness of summarized estimate was shown by the data above.

## Discussion

OA, known as a degenerative disease in the aging population, is the most universal cause of joint disease which could finally result in physical disability [23]. Although OA is considered as a multifactorial disease, genetic factors are reported as vital determinants in the pathogenesis of this disorder [6, 7]. Enormous attention has been paid to the association between gene SNPs and risk of osteoarthritis, and SMAD3 SNP rs12901499 was studied by several researchers. In different studies, the results ranged from no association to the strong linkage between the SNPs and the disorder [12, 14, 16, 17]. The inconsistent findings on the associations between OA and SMAD3 rs12901499 polymorphism in Asians from relatively underpowered studies above may be attributed to factors like small sample size and different population. So we conducted this systematic review and meta-analysis to draw a more definitive conclusion.

To obtain compelling evidence of the linkage between SMAD3 rs12901499 polymorphism and risk of OA, we enrolled a total of 5093 OA patients and 5699 controls from 10 studies in this meta-analysis. The pooled results showed there is an association between OA and rs12901499 polymorphism in the overall population. And subgroup analysis stratified by ethnicity demonstrated that G allele increased the risk of OA in Caucasian. While in Asians, no association was found between SMAD3 rs12901499 polymorphism and OA risk. The results seem intriguing that there is association in the overall population but without positive result in Asian. It could be probably statistically insufficient when the majority of cases of the study were originated from Caucasian (6100/10792). And one other possibility may relate to the different expression patterns of Smad3 in non-homogeneous ethnic populations.

Sensitivity analysis and bias estimation warrant the stability of our meta-analysis.

Based on the present analysis, we propose that patients harboring the G allele of SMAD rs12901499 polymorphism experience an increased susceptibility to OA in Caucasian, though the mechanism underlying the association between SMAD3 rs12901499 polymorphism and osteoarthritis is temporarily unknown. It was reported that SMAD2/3 molecules, known as a part of the TGF-B signaling, have been linked to the chondrocyte anabolism. Previous study showed mutation of SMAD3 could lead to lower expression of type II collagen [17]. Through an in vivo study, Wu et al. found that loss of Smad3 could enhance the BMP signaling to induce articular chondrocytes hypertrophy and lose its normal phenotype [24]. Further functional studies are required to identify the role of SNP rs12901499 in OA susceptibility.

The meta-analysis presented does have some limitations. Firstly, the genotype distribution data was not available in Valdes’ study and only allele model among overall population was analyzed to assess the association. Secondly, OA was considered as a multifactorial disease; however, the interactions between the gene and environment were not fully addressed in this meta-analysis, which may magnify the role of Smad3 polymorphism in osteoarthritis. Thirdly, heterogeneity, which comes from different designation about interventions, participants, or outcomes in amount of studies, was inevitably existed though partially addressed by subgroup analysis stratified by ethnic groups and OA sites. Fourthly, since the data is not completely available, the subgroup analysis stratified by every potential confounders in including BMI, age, and gender could not be performed, which may lead to relatively inaccurate pooled results. Last, though there was no obvious publication bias revealed by funnel plot and Egger’s test, the selection bias could not be completely removed because only studies published in English were included.

## Conclusion

In conclusion, the present meta-analysis demonstrated that the G variant of SMAD3 rs12901499 polymorphism increased the risk of OA in Caucasians. By contrast, SMAD3 rs12901499 polymorphism was not associated with OA risk in Asians. Due to the limitations of our study, further well-designed studies with larger sample size in different ethnic populations should be performed to confirm these results.

## Additional files

Additional file 1: **Table S1.** PRISMA checklist. (DOC 63 kb)

Additional file 2: **Table S2.** Results of quality assessment for the included studies using the Newcastle–Ottawa Scale. (DOCX 21 kb)

## Abbreviations

OA: Osteoarthritis
OR: Odds ratio
SMAD3: SMAD family member 3
SNP: Single nucleotide polymorphisms
TGF: Transforming growth factor
