# Supplementary material for: Association between SMAD3 gene polymorphisms and osteoarthritis risk: a systematic review and meta-analysis
Source: J Orthop Surg Res. 2018 Sep 12;13:232. doi: 10.1186/s13018-018-0939-2 (PMC6134766; doi:10.1186/s13018-018-0939-2)
Supplement: Supplementary file 2 — Table S2. Results of quality assessment for the included studies using the Newcastle–Ottawa Scale. (DOCX 21 kb) [file 13018_2018_939_MOESM2_ESM.docx]

**Table S2.** Results of quality assessment for the included studies using the Newcastle–Ottawa Scale

| Study | Selection | | | |  | Comparability |  | Exposure/Outcome | | | Scores |
| --- | --- | --- | --- | --- | --- | --- | --- | --- | --- | --- | --- |
|  | Q1 | Q2 | Q3 | Q4 |  | Q5 ^a^ |  | Q6 | Q7 | Q8 |  |
| **Cohort studies** |  |  |  |  |  |  |  |  |  |  |  |
| Valdes-Chingford | **☆** | **☆** | **☆** | **☆** |  | **☆☆** |  | **☆** | **☆** | **☆** | 9 |
| Valdes-Hertfordshire | **☆** | **☆** | **☆** | **☆** |  | **☆** |  | **☆** | **☆** | **☆** | 8 |
| Valdes-Estonia | **☆** | **☆** | **☆** | **☆** |  | **☆** |  | **☆** | **☆** | **☆** | 8 |
| **Case-control studies**  Valdes-Discovery set | **☆** | **☆** | **-** | **☆** |  | **☆** |  | **☆** | **☆** | **☆** | 7 |
| Valdes-Nottingham | **☆** | **☆** | **-** | **☆** |  | **☆** |  | **☆** | **☆** | **☆** | 7 |
| Jiang | **☆** | **☆** | **☆** | **☆** |  | **-** |  | **☆** | **☆** | **☆** | 7 |
| Su | **☆** | **☆** | **☆** | **☆** |  | - |  | **☆** | **☆** | **☆** | 7 |
| Sharma | **☆** | **☆** | **-** | **☆** |  | **☆☆** |  | **☆** | **☆** | **☆** | 8 |
| Zhang | **☆** | **☆** | **☆** | **☆** |  | **☆** |  | **☆** | **☆** | **☆** | 8 |
| Zhong | **☆** | **☆** | **☆** | **-** |  | **☆☆** |  | **☆** | **☆** | **☆** | 8 |

^a^ A maximum of 2 stars can be allotted in this category, one for age, the other for other controlled factors.

**NEWCASTLE - OTTAWA QUALITY ASSESSMENT SCALE**

**FOR CASE CONTROL STUDIES**

Note: A study can be awarded a maximum of one star for each numbered item within the Selection and Exposure categories. A maximum of two stars can be given for Comparability

**Selection**

Q1) Is the case definition adequate?

a) yes, with independent validation **🟑**

b) yes, eg record linkage or based on self reports

c) no description

Q2) Representativeness of the cases

a) consecutive or obviously representative series of cases **🟑**

b) potential for selection biases or not stated

Q3) Selection of Controls

a) community controls **🟑**

b) hospital controls

c) no description

Q4) Definition of Controls

a) no history of disease (endpoint) **🟑**

b) no description of source

**Comparability**

Q5) Comparability of cases and controls on the basis of the design or analysis

a) study controls for the most important factor. **🟑**

b) study controls for any additional factor. **🟑**

**Exposure**

Q6) Ascertainment of exposure

a) secure record (eg surgical records) **🟑**

b) structured interview where blind to case/control status **🟑**

c) interview not blinded to case/control status

d) written self report or medical record only

e) no description

Q7) Same method of ascertainment for cases and controls

a) yes **🟑**

b) no

Q8) Non-Response rate

a) same rate for both groups **🟑**

b) non respondents described

c) rate different and no designation

**NEWCASTLE - OTTAWA QUALITY ASSESSMENT SCALE**

**FOR COHORT STUDIES**

Note: A study can be awarded a maximum of one star for each numbered item within the Selection and Outcome categories. A maximum of two stars can be given for Comparability

**Selection**

Q1) Representativeness of the exposed cohort

a) truly representative of the average population in the community **🟑**

b) somewhat representative of the average population in the community

c) selected group of users

d) no description of the derivation of the cohort

Q2) Selection of the non exposed cohort

a) drawn from the same community as the exposed cohort **🟑**

b) drawn from a different source

c) no description of the derivation of the non-exposed cohort

Q3) Ascertainment of exposure

a) secure record **🟑**

b) structured interview **🟑**

c) written self-report

d) no description

Q4) Demonstration that outcome of interest was not present at start of study

a) yes *

b) no

**Comparability**

Q5) Comparability of cohorts on the basis of the design or analysis

a) study controls for the most important factor. **🟑**

b) study controls for any additional factor. **🟑**

**Outcome**

Q6) Assessment of outcome

a) independent blind assessment **🟑**

b) record linkage **🟑**

c) self-report

d) no description

Q7) Was follow-up long enough for outcomes to occur

a) yes **🟑**

b) no

Q8) Adequacy of follow up of cohorts

a) complete follow up - all subjects accounted for **🟑**

b) subjects lost to follow up unlikely to introduce bias - small number lost > 70 % follow up, or description provided of those lost

c) follow up rate < 70% and no description of those lost

d) no statement
